# Approaches to increase recovery of bacterial and fungal abortion agents in domestic ruminants

**DOI:** 10.4102/ojvr.v90i1.2010

**Published:** 2023-01-11

**Authors:** Annelize Jonker, Peter N. Thompson, Anita L. Michel

**Affiliations:** 1 Tuberculosis and Brucellosis Research Programme, Department of Veterinary Tropical Diseases, Faculty of Veterinary Science, University of Pretoria, Pretoria, South Africa; 2 Department of Production Animal Studies, Faculty of Veterinary Science, University of Pretoria, Pretoria, South Africa

**Keywords:** abortion, culture, *Brucella*, *Campylobacter*, bovine, ovine, caprine, ruminants

## Abstract

Abortions in domestic ruminants cause significant economic losses to farmers. Determining the cause of an abortion is important for control efforts, but it can be challenging. All available diagnostic methods in the bacteriology laboratory should be employed in every case due to the many limiting factors (autolysis, lack of history, range of samples) that complicate the investigation process. The purpose of this study was to determine whether the recovery of diagnostically significant isolates from domestic ruminant abortion cases could be increased through the use of a combination of the existing aerobic culture and *Brucella* selective method with methods that are commonly recommended in the literature reporting abortion investigations. These methods are examination of wet preparations and impression smears stained by the modified Ziehl–Neelsen method, anaerobic, microaerophilic, *Leptospira, Mycoplasma* and fungal culture. Samples of placenta and aborted foetuses from 135 routine clinical abortion cases of cattle (*n* = 88), sheep (*n* = 25) and goats (*n* = 22) were analysed by the new combination of methods. In 46 cases, bacteria were identified as aetiological agents and in one case a fungus. Isolation of *Brucella* species increased to 7.4% over two years compared with the previous 10 years (7.3%), as well as *Campylobacter jejuni* (*n* = 2) and *Rhizopus* species (*n* = 1). *Salmonella* species (5.9%) and *Trueperella pyogenes* (4.4%) were also isolated more often. In conclusion, the approach was effective in removing test selection bias in the bacteriology laboratory. The importance of performing an in-depth study on the products of abortion by means of an extensive, combination of conventional culture methods was emphasised by increased isolation of *Brucella abortus* and isolation of *C. jejuni*. The combination of methods that yielded the most clinically relevant isolates was aerobic, microaerophilic, *Brucella* and fungal cultures.

## Introduction

Abortions in domestic ruminants cause significant economic losses to farmers worldwide (Borel et al. 2014). Abortion is defined as the expulsion of the foetus from the uterus before it is full term and viable (Baumgartner 2015). Diagnosis of the cause of an abortion plays an important role in the subsequent mitigation and control efforts (Borel et al. 2014). A diagnosis depends not only on the detection of a bacterial/fungal agent but also on finding macro- and/or microscopic lesions indicative of bacterial/fungal infection in foetal tissues, which can be challenging (Clothier & Anderson 2016; Wheelhouse & Dagleish 2014). Autolysis is one of the most important factors the laboratory diagnostician has to contend with, because it masks lesions and the agent of abortion may be outcompeted by contaminating bacteria (Kirkbride 1986). Foetuses may already be autolytic by the time they are aborted because of an infection process or maceration (Clune et al. 2021; Givens & Marley 2008). Other major stumbling blocks tend to be lack of history and incomplete range of samples received at the laboratory (Holler 2012; Wheelhouse & Dagleish 2014). Limited test selection, a cost-reducing measure, often aimed at detecting the most common agents or those that can be zoonotic can drastically reduce the chances of reaching a diagnosis (Borel et al. 2014; Schnydrig et al. 2017).

Placenta and abomasal fluid samples are most likely to yield the organism of interest (Clothier & Anderson 2016; Holler 2012). Agents of abortion that only affect the placenta such as *Chlamydia* species, *Coxiella burnetii*, as well as *Bacillus licheniformis* and some fungi, may not be detected if the placenta is not included (Clothier & Anderson 2016; Wheelhouse & Dagleish 2014). Fungal infections may result in macroscopic lesions in the placenta but not in the foetus (Borel et al. 2014). Kidney and liver samples are useful for the detection of *Leptospira interrogans* (eds. Markey et al. 2013; Mgode et al. 2015).

The use of dark field microscopy of wet mounts as well as impression smears was reported by several studies. Kirkbride (1992) as well as Macías-Rioseco et al. (2020) used dark field microscopy to screen for motile bacteria, such as *Campylobacter*, in abomasal fluid. Impression smears stained by the modified Ziehl–Neelsen method (MZN) were included in routine abortion investigations although the method was shown to have limited sensitivity and specificity (Borel et al. 2014; Schnydrig et al. 2017). In a South African report of chlamydiosis in a beef herd, Ehret et al. (1975) mentioned the use of the modified Ziehl–Neelsen method.

Conventional culture methods vary between laboratories, different species of animals and countries. Bacterial isolation employing aerobic, as well as microaerophilic culture was commonly reported (Agerholm et al. 2006; Campero et al. 2003; Clothier & Anderson 2016; Schnydrig et al. 2017). Aerobic culture on agar plates with 5% blood in a 5% CO_2_ atmosphere is sufficient to isolate most bacteria (Borel et al. 2014). Addition of microaerophilic culture increases sensitivity for bacteria that are sensitive to oxygen (Borel et al. 2014). Anaerobic bacteria such as *Fusobacterium necrophorum, Fusobacterium nucleatum* and *Bacteroides fragilis* have been reported in cases of ruminant abortion on rare occasions (Agerholm et al. 2006; Syrjälä et al. 2007), which may be ascribed to the fact that anaerobic culture is not commonly included in abortion panels (Borel et al. 2014). Schnydrig et al. (2017) included selective media for *Brucella, Campylobacter, Listeria* and fungi to increase the chances of isolating these microorganisms by suppressing the growth of contaminating bacteria.

A number of bacterial agents of abortion are contagious and/or become endemic in herds. *Brucella abortus* (*B. abortus*) is common and *Brucella melitensis* (*B. melitensis*) as well as *Brucella ovis* (*B. ovis*) are isolated sporadically in South Africa (Kolo et al. 2019; Matle et al. 2021; Mbizeni 2015). *Campylobacter fetus* ssp. *venerealis, Campylobacter fetus* ssp. *fetus, Campylobacter jejuni* and *Arcobacter* species are occasionally implicated in cases of abortion in South Africa (Bath et al. 2013; Coetzer & Oberem 2018), while *Campylobacter* species were more commonly reported as causes of abortion in Argentina and New Zealand (Campero et al. 2003; West et al. 2002). The families Chlamydiaceae, Waddliaceae, Parachlamydiaceae and Rhabdochlamydiaceae are of significance in contagious abortions (Borel et al. 2014). However, members of these families fall in the difficult to culture category, because they do not grow on conventional growth media (Vidal et al. 2017). Serological studies indicate that *Chlamydia abortus* plays a role in ruminant abortions in South Africa (Ndou & Dlamini 2012; Sri Jeyakumar 2001). *L. interrogans* serovars have been implicated in bovine abortion in South Africa and other countries such as the United States (Clothier & Anderson 2016; Herr et al. 1982; Knudtson & Kirkbride 1992). Several *Salmonella* subspecies and serovars are associated with abortion in ruminants, for example, *Salmonella enterica* subspecies *diarizonae, Salmonella enterica* subspecies *enterica* serovar Dublin and *Salmonella enterica* subspecies *enterica* serovar Abortusovis (eds. Markey et al. 2013). *Salmonella enterica* subspecies *enterica* serovar Dublin is a known cause of abortions in cattle in South Africa (Coetzer & Oberem 2018).

Opportunistic bacterial agents of abortion may be commensals of mucous membranes or may be common in the environment, for example, *Bacillus* spp., *Escherichia coli* (*E. coli*), *Histophilus somni, Pasteurella* spp., *Staphylococcus* spp., *Streptococcus* spp., *Trueperella pyogenes* (*T. pyogenes*) and *Mycoplasma* species. Abortions caused by these bacteria are sporadic, but their occurrence is widespread (Clothier & Anderson 2016).

Mycotic or fungal abortion is caused by opportunistic fungal pathogens that are normally present as saprophytes in the environment of the animal. Fungi most commonly associated with abortion are *Aspergillus* species and *Candida* species (Anderson 2007; Holler 2012; Mee 2020).

Additional herd information and history such as the number of abortions, the number of pregnant animals, clinical signs in dams and changes in husbandry are useful (Borel et al. 2014). Number of abortions per number of pregnant animals (abortion rate) is a useful guideline to determine whether an investigation is indicated or not. Abortion rates less than 2% are usually not seen as an indication for investigation (Menzies 2011). Most abortions are sporadic with less than 5% of pregnant animals aborting (Givens & Marley 2008). However, it must be borne in mind that an abortion rate of between 2% and 5% may be an indication that an endemic infectious agent is present (Menzies 2011). Additional information must be interpreted with caution as it can be misleading. For example, aetiological agents should not be ruled out simply because animals were vaccinated as vaccination does not result in complete immunity (Kirkbride 1986). Moreover, unvaccinated, infected animals could have been introduced into a herd prior to an abortion event.

The aim of this study was to analyse samples of placenta and aborted foetuses from cattle, sheep and goats by means of a set combination of conventional culture and identification methods for bacteria and fungi. The objective was to determine whether more diagnostically significant isolates could be cultured if anaerobic, *Campylobacter, Leptospira, Mycoplasma* and fungal culture were added to the existing aerobic and *Brucella* selective culture and if applying the new combination of methods to every abortion case could remove test selection bias.

## Materials and methods

Routine diagnostic samples from products of abortion from cattle, sheep and goats submitted to the bacteriology laboratory of the Department of Veterinary Tropical Diseases, Faculty of Veterinary Science, University of Pretoria over a two-year period (September 2017 to September 2019) were included in the study. Samples were accepted from the Pathology section at the Faculty of Veterinary Science, regional government laboratories, private laboratories, private veterinarians or State veterinarians. Criteria for selection was abortion meaning a foetus expelled from the uterus before it was full term and viable (Baumgartner 2015). Indications that the calf/lamb/kid was born alive such as aerated lungs, colostrum in the abomasum or worn golden slippers were criteria for exclusion. One or more foetuses submitted as a single case were recorded as a single case, except when different agents were recovered from the different foetuses. Samples accepted were any of the following: placenta, stomach content, lung, liver, spleen and kidney from aborted foetuses. Samples were accepted whether they were in good or poor condition.

Sample size was calculated as 235 foetuses for this descriptive study (Survey toolbox-www.epitools.ausvet.com.au) (Fosgate 2009); therefore, the aim was to analyse samples from 200 to 300 foetuses.

### Conventional microbiological analyses

The methods that were previously applied to placenta and foetus samples in the bacteriology laboratory at the DVTD were impression smears stained by the Gram and modified Ziehl–Neelsen methods as well as aerobic culture, *Brucella* selective culture and fungal culture. *Brucella* selective culture and fungal culture were performed on request. For the purpose of this study, wet mounts, microaerophilic culture, anaerobic culture, *Mycoplasma* selective culture and *Leptospira* selective culture were added.

### Direct smears

Wet mounts were made of stomach content samples, if included in abortion cases, and examined by dark field microscopy for *Campylobacter* species, *Leptospira* species and other motile bacteria. Impression smears were made of all placenta and foetus samples, heat fixed and stained by Gram and MZN methods (eds. Markey et al. 2013).

### Aerobic culture

All samples were inoculated on Columbia blood agar with 5% horse blood (CBA) (Selecta-media, Thermofischer, Gauteng, South Africa), MacConkey agar (McC) (without crystal violet) (Selecta-media, Thermofischer, Gauteng, South Africa) and Xylose-Lactose-Desoxycolate medium (XLD) (Oxoid CM0469B, Thermofischer, Gauteng, South Africa). Inoculated CBA plates were incubated in a 5% carbon dioxide (CO_2_) atmosphere at 37 ºC ± 1 ºC and McC and XLD plates were incubated in normal air at 37 ºC ± 1 ºC. CBA plates were examined daily for 10 days. McC plates were examined after 24 h. Primary identification consisted of Gram stain as well as catalase and oxidase tests. The outcome of these tests determined carbohydrate combinations for secondary identification (eds. Markey et al. 2013).

### Anaerobic culture

Only stomach content was inoculated on pre-reduced CBA and incubated anaerobically at 37 ºC ± 1 ºC for 5 days in an anaerobic workstation (Bactron anaerobic chamber, United Scientific, Gauteng, South Africa). If stomach content was not received, this culture method was not performed. An anaerobic detector strip (Oxoid, Thermoscientific, Gauteng, South Africa) was used to confirm anaerobiosis. Primary identification consisted of Gram stain, catalase and oxidase tests. Depending on the outcome of these tests, isolates were identified by Mastring (Mast ID, Davies Diagnostics, Gauteng, South Africa) or Rapid ID 32A (Biomerieux, Gauteng, South Africa) (eds. Markey et al. 2013).

### Brucella selective culture

In addition to aerobic culture, placenta, stomach content and/or lung were inoculated on Farrell medium (Oxoid CM0169 with supplement (SR0083A), Thermoscientific, Gauteng, South Africa). If these samples were not received, this culture method was not performed. One plate of Farrell medium was inoculated with an in-house *B. abortus* control culture for quality control purposes. Plates were incubated in CO_2_ at 37 °C ± 1 °C for 10 days and examined daily. Cultures were regarded as negative when no characteristic growth was obtained by day 10. Suspect colonies were subcultured onto two CBA plates and one McC plate. One CBA plate was incubated in CO_2_, the other CBA plate was incubated in normal air together with the McC plate. Primary identification included a Gram stain, oxidase, MZN stain and a Urease slant (Selecta-media, Thermoscientific, Gauteng, South Africa) (WOAH 2016). Presumptive *Brucella* species identified were submitted to the ARC-OVR for confirmation and phenotyping.

### Salmonella selective culture

In addition to direct culture for *Salmonella*, placenta, stomach content and organ samples were inoculated on Rappaport-Vassiliades Broth (RV) (Oxoid CM 0866B, Thermoscientific, Gauteng, South Africa) and incubated at 42 °C ± 1 °C in normal air. After 24 h, the broth culture was inoculated on XLD medium (Oxoid CM0469B, Thermoscientific, Gauteng, South Africa) and incubated at 37 °C ± 1 °C in normal air. Primary identification included a Gram stain, catalase, oxidase and spot indole tests. API^®^ 10S (Biomerieux, Gauteng, South Africa) was used for secondary identification (eds. Markey et al. 2013). If no characteristic colonies were seen, the culture was regarded as negative. *Salmonella* isolates were serotyped according to the White-Kauffman-Le Minor scheme (Grimont & Weil 2007).

### Mycoplasma selective culture

In addition to aerobic culture, stomach content, placenta and lung were inoculated directly on a plate of *Mycoplasma* medium (Oxoid CM0401 & SR0059, Thermoscientific, Gauteng, South Africa) and in *Mycoplasma* broth (Oxoid CM0403 & SR0059, Thermoscientific, Gauteng, South Africa). If these samples were not received, this culture method was not performed. The broth was incubated in normal air at 37 ºC ± 1 ºC and plated onto *Mycoplasma* medium after 24 h. Plates were incubated in CO_2_ at 37 ºC ± 1 ºC; examined at 48 and 96 h after inoculation and thereafter once a week. If no characteristic growth was obtained after 14 days’ incubation, the culture was regarded as negative. Suspect cultures were identified to genus level as described by Markey et al. (eds. 2013).

### Campylobacter culture

Placenta, stomach content and liver were inoculated on CBA. If none of these samples were received, this culture method was not performed. Inoculated plates were incubated in a microaerophilic atmosphere (6% O_2_, 10% CO_2_ and 84% N) (CampyGen, Oxoid Ltd, Thermoscientific, Gauteng, South Africa) in anaerobic jars (Oxoid Ltd, Thermoscientific, Gauteng, South Africa) at 37 ºC ± 1 ºC for five days. The plates were examined after 72 h and again at the end of five days. If no suspect cultures were noted by day five, plates were discarded as negative. Primary identification included Gram stain, catalase and oxidase tests. Secondary identification included growth in a microaerophilic atmosphere at 42 ºC, growth at 37 ºC in normal air, growth at 22 ºC, growth in the presence of 1% glycine, production of H_2_S in triple sugar iron medium (TSI) (Selecta-media, Thermoscientific, Gauteng, South Africa) and hippurate hydrolysis (Remel, Thermoscientific, Gauteng South Africa) (eds. Markey et al. 2013).

Charcoal-Cefoperazone-Dextrose-Agar (CCDA) (Selecta media, Thermoscientific, Gauteng, South Africa) was inoculated with placenta, stomach content and liver samples from cases that yielded *Campylobacter* isolates on CBA. The *Campylobacter* isolates from these cases and a control strain, *C. jejuni* (ATCC 33560) were also inoculated on CCDA. The inoculated medium was incubated in a microaerophilic atmosphere at 37 ºC ± 1 ºC for five days. The plates were examined for suspect colonies after 72 h and again after five days. If no suspect colonies were observed by day five, plates were discarded as negative. Primary and secondary identification of suspect colonies was performed as described above.

### Leptospira culture

One tube of *Leptospira* Ellinghausen–McCullough–Johnson–Harris (EMJH) medium (Difco 279410 & supplement 279510, Becton Dickinson, Gauteng, South Africa) was inoculated with 1 g kidney or liver. If none of these samples were received, this culture method was not performed. Cultures were incubated at 30 ºC ± 1 ºC in normal air and examined weekly for 13 weeks. Contaminated cultures were subcultured into a new tube of EMJH medium with 5’-fluorouracil (Sigma, Gauteng, South Africa). Turbidity and/or the formation of Dinger’s ring as well as characteristic morphology on dark-field microscopy was regarded as presumptive positive. If no turbidity is evident by week 13, cultures were regarded as negative (eds. Markey et al. 2013). Presumptive positive cultures were submitted to the Agricultural Research Council-Onderstepoort Veterinary Research (ARC-OVR) for polymerase chain reaction (PCR) confirmation.

### Fungal culture

Stomach content and placenta (as well as skin lesions, if submitted) were inoculated on Potato Dextrose agar (Selecta-media, Thermoscientific, Gauteng, South Africa). If none of these samples were received, this culture method was not performed. The plates were incubated at 37 ºC for five days. Plates were examined twice a week. A sticky tape colony smear was made of suspect colonies and stained by Lactophenol Cotton Blue stain (Merk, Gauteng, South Africa). Fungi were identified by colony morphology. Yeasts were identified by Gram stain and API^®^ 20C AUX (Biomerieux, Gauteng, South Africa) (eds. Markey et al. 2013).

### Supplementary information

Data such as pathology lesions, immunohistochemical staining results and PCR results were extracted from diagnostic reports for supplementary information. The methodologies for these procedures were, however, not available.

### Herd history

Additional production data such as farming system, feeding system, number of pregnant animals, number of abortions and vaccination status were extracted from pathology reports.

### Data capture and analysis

Data were captured in an Excel spreadsheet from which summary tables were created. The aim was to investigate patterns with respect to diagnostic rate, completeness of submission, condition of samples, methods used and season. Diagnostic rate was calculated by dividing the number of confirmed diagnoses by the total number of submissions and multiplying by 100. The result was expressed as a percentage. A confirmed diagnosis was defined as a diagnosis made by the pathologist taking into account the pathological lesion and the bacterial or fungal isolate. Abortion rate was calculated by dividing the number of abortions by the number of pregnant animals in a herd (if available) and multiplying by 100. The result was expressed as a percentage. Abortion rate is used to determine the significance of the number of abortions in a herd. Data were exported into a statistical program (Epi info 7™ 7.2.3.1, Centers for Disease Control and Prevention). Percentages with 95% confidence intervals (CI) were calculated, and multivariate logistic regression was used to investigate associations between completeness of submission, condition of samples, confirmed diagnosis and season.

### Ethical considerations

Ethical clearance to conduct this study was obtained from the University of Pretoria, Faculty of Veterinary Science Research Ethics and Animal Ethics Committee. (No. V092-17 & V070-21).

## Results

### Number of cases

The calculated number of 235 abortion cases was not reached because of lower-than-expected submission rates during 2018 and 2019. Samples from 135 abortion cases were received for bacterial and fungal culture. Eighty-eight were bovine, 25 were ovine and 22 were caprine cases. Forty-six complete submissions (foetus and placenta) were received: 26 bovine, 11 ovine and nine caprine cases. Eighty-nine submissions were incomplete: 85 cases of foetus only and four cases of placenta only. Most samples (*n* = 100) were considered to be good quality and 35 were poor quality (Table 1).

**TABLE 1:** Diagnosis and diagnostic rate (with 95% confidence intervals) compared to the completeness of submissions as well as quality of samples.

Submission	Number of submissions	Confirmed diagnosis (diagnostic rate)			Quality of samples	Number	Confirmed diagnosis (diagnostic rate)		
		*n*	%	95% Cl			*n*	%	95% Cl
Foetus and placenta	46	20	43.5	13, 27	Good	33	15	45.5	9, 21
					Poor	13	5	38.5	2, 9
Foetus only	85	36	42.4	27, 46	Good	63	31	49.2	23, 39
					Poor	22	5	22.7	2, 10
Placenta only	4	1	25.0	0, 3	Good	4	1	25.0	0, 3

**Total**	**135**	**57**	**42.2**	**46, 69**	-	-	-	-	-

95% CI, 95% confidence interval.

### Diagnostic rate

Overall diagnostic rate for cases submitted to the bacteriology laboratory was 42.2% (95% CI: 46, 69). Diagnostic rate per animal species was 36 of 88 bovine cases (32.9%, 95% CI: 27, 46), 14 of 25 ovine cases (48.0%, 95% CI: 9, 19) and seven of 22 caprine cases (27.3%, 95% CI: 3, 12). Bovine cases were most often received (88/135). Complete submissions (foetus and placenta) had the highest diagnostic rate overall at 43.5% (95% CI: 13, 27) although this was not the case for all species. Diagnostic rate for complete bovine cases was 10 of 26 (38.5%, 95% CI: 5, 15) and for incomplete cases 24 of 62 (38.7%, 95% CI: 16, 32). Diagnostic rate for complete ovine cases was five of 11 (45.5%, 95% CI: 2, 8) and for incomplete cases seven of 14 (50%, 95% CI: 3, 11). The diagnostic rate for complete caprine cases was three of nine (33.3%, 95% CI: 1, 6) and for incomplete cases, three of 13 (23%, 95% CI: 1, 7). Submissions where the placenta was included (*n* = 50) had a diagnostic rate of 42.0% (95% CI: 14, 28). In 47 of 57 submissions where a diagnosis was made, bacteria or fungi were implicated (Table 1).

Most cases were submitted in winter (*n* = 59) followed by spring (*n* = 34). Most *B. abortus*-positive cases (*n* = 8) and *T. pyogenes*-positive cases (*n* = 6) were submitted in winter and spring.

Multivariate logistic regression analysis was performed to investigate the association between diagnosis and degree of autolysis, completeness of submission as well as season (Table 2).

**TABLE 2:** Multivariate regression analysis investigating association between diagnosis and degree of autolysis, completeness of submission and season.

Term	Odds ratio	95% CI	Coefficient	s.e.	*Z*-statistic	*p*-value
Autolysis (yes/no)	11 215	0.454, 27 704	0.1146	0.4614	0.2484	0.8038
Complete submission (yes/no)	13 444	0.6393, 28 272	0.2960	0.3793	0.7804	0.4352
Season (spring/autumn)	18 074	0.4681, 69 785	0.5919	0.6893	0.8587	0.3905
Season (spring/autumn)	19 606	0.4737, 81 152	0.6733	0.7247	0.9289	0.3529
Season (spring/autumn)	16 928	0.4787, 5986	0.5264	0.6444	0.8169	0.4140

Note: Constant: Coefficient = –11 723, standard error = 0.6236, *Z*-statistics = –1.88, *p* = 0.0601. Convergence = converged; Iterations = 3; Final –2*Log-likelihood = 1 764 165; Cases included = 135.

Score: statistic = 15 222, degree of freedom = 5, *p* = 0.9105. Likelihood ratio: statistics = 15 547, degree of freedom = 5, *p* = 0.9067.

95% CI, 95% confidence interval; s.e., standard error.

The odds ratio and CI for autolysis, complete submission and season are > 1, but *p* > 0.05. This indicates that there is no significant association between diagnosis and degree of autolysis, complete submission (inclusion of placenta) or season.

No wet mounts were positive for motile bacteria. Modified Ziehl–Neelsen stained impression smears were positive for small, partially acid-fast coccobacilli in six of 10 cases (60%, 95% CI: 3, 9) that yielded *B. abortus*-positive cultures. In addition, three cases that were positive for partially acid-fast rods on stained impression smears were PCR positive for *C. burnetii*. No Chlamydiales-positive cases were positive for partially acid-fast bacteria on impression smears.

Eighty-five potentially abortifacient isolates were cultured from 135 submissions. Bacteria were most common (73/85). Twelve isolates were endemic/contagious bacteria and 73 were environmental or commensal bacteria that cause sporadic abortions. Twelve isolates were fungi. In 46 cases, bacteria were identified as aetiological agents and in one case a fungus (Table 3). *B. abortus* was the most common of the endemic/contagious bacteria at 10/12 isolates. It was also one of the most common bacterial isolates overall (10/71).

**TABLE 3:** Significant bacterial and fungal isolates from products of abortion detected by impression smears stained by the Modified Ziehl–Neelsen method, conventional culture, immunohistochemistry and PCR.

Case no.	Pathology lesions	Bacterial/fungal isolate	Detection method			
			MZN	Culture	IHC	PCR
**Bovine abortions**						
1	Purulent bronchointerstitial pneumonia	*Trueperella pyogenes*	X	A	ND	ND
2	No specific lesions (No placenta)	*Trueperella pyogenes*	N	A	ND	ND
3	Meningitis	*Brucella abortus* biovar 1	X	A, B	ND	ND
4	Meningitis, bronchointerstitial pneumonia	*Brucella abortus* biovar 1	X	A, B	ND	ND
5	Non-specific lesions (No placenta)	*Campylobacter fetus* spp. *venerealis* biovar *intermedius*	N	A, M	ND	ND
6	Purulent bronchopneumonia	*Trueperella pyogenes*	N	A	ND	ND
7	Non-specific lesions (No placenta)	*Salmonella* sp.	N	A	ND	ND
8	Necropurulent placentitis	*Trueperella pyogenes*	N	A	ND	ND
9	Purulent bronchopneumonia	*Brucella abortus* biovar 1	X	A, B	ND	ND
10	Necrotic placentitis, purulent pneumonia	*Chlamydia* species	N	ND	X	ND
		*Salmonella* sp.	N	A	ND	ND
11	Purulent bronchopneumonia	*Trueperella pyogenes*	N	A	ND	ND
12	Bronchointerstitial pneumonia	*Brucella abortus* biovar I	X	A, B	ND	ND
13	No specific lesions (No placenta)	*Salmonella* sp.	N	A	ND	ND
14	Necrotic placentitis with fungal hyphae, purulent bronchopneumonia	*Bacillus licheniformis*	N	A	ND	ND
		*Rhizopus* sp.	N	F	ND	ND
15	Necrotic placentitis	*Streptococcus dysgalactiae*	N	A	ND	ND
		*Acholeplasma* sp.	N	M	ND	ND
16	Pulmonary and hepatic congestion	*Brucella abortus* biovar I	N	A, B	ND	ND
		*Acholeplasma* sp.	N	M	ND	ND
17	Generalised congestion, hepatomegaly, bronchopneumonia	*Brucella abortus* biovar 1	N	A, B	ND	ND
		*Salmonella* sp.	N	A	ND	ND
18	Yellow liver	*Salmonella* sp.	N	A	ND	ND
19	Neutrophilic interstitial pneumonia	*Pseudomonas luteola*	N	A	ND	ND
20	Necrotic placentitis	*Campylobacter fetus*	N	A, C	ND	ND
		*Mannheimia varigena, Streptococcus pluranimalium*	N	A	ND	ND
		*Mycoplasma* sp.	N	M	ND	ND
21	Necrotic placentitis and vasculitis	*Mycoplasma* sp.	N	C	ND	ND
22	S/C oedema, hydrothorax, ascites,	*Trueperella pyogenes*	N	A	ND	ND
23	Necrotic placentitis with vasculitis	*Coxiella burnetii*	X	ND	X	ND
24	Interstitial pneumonia	*Brucella abortus* biovar 1	N	A, B	ND	ND
25	Purulent bronchopneumonia	*Aeromonas hydrophila*	N	A	ND	ND
26	Hepatitis, vasculitis, bacteria in organs	*Staphylococcus delphini*	N	A	ND	ND
27	Bronchopneumonia, hepatic necrosis	*Brucella abortus* (Field strain)	N	A, B	ND	ND
28	Purulent bronchopneumonia	*Brucella abortus* biovar 2	X	A, B	ND	ND
29	No lesions (No placenta)	*Brucella abortus* biovar 1	X	A, B	ND	ND
**Caprine abortions**						
30	Meningoencephalitis	*Campylobacter jejuni* ssp. *jejuni*	N	C	ND	ND
31	Lung haemorrhages, hepatic necrosis	*Salmonella enterica* ssp. *arizonae*	N	A	ND	ND
32	Necrotic placentitis	*Coxiella burnetii*	X	ND	X	ND
		*Salmonella typhimurium*	N	A	ND	ND
33	No lesions (No placenta)	*Chlamydia abortus*	N	ND	ND	X
		*Chlamydia pecorum*				
34	Brain congestion, severe interstitial pneumonia	*Escherichia coli*	N	A	ND	ND
35	Bacteraemia	*Escherichia coli*	N	A	ND	ND
**Ovine abortions**						
36	Necrotic, purulent placentitis Bronchopneumonia	*Chlamydia abortus*	N	ND	ND	X
		*Escherichia coli*	N	A	ND	ND
37	Purulent bacterial placentitis	*Escherichia coli*	N	A	ND	ND
38	Congestion of placenta, brain, liver; lymphoplasmacytic infiltration of myocardium	*Salmonella* D	N	A	ND	ND
39	Non-specific lesions (No placenta)	*Chlamydia* sp.	N	ND	X	ND
		*Salmonella* sp.	N	A	ND	ND
40	Pleuropneumonia, bacterial and fungal placentitis	*Chlamydia abortus*	N	ND	ND	X
41	Bacterial placentitis	*Arcobacter* sp.	N	A	ND	ND
42	Necrotic placentitis	*Salmonella* Budapest	N	A	ND	ND
		*Mycoplasma* sp.	N	M	ND	ND
43	Necrotic placentitis, purulent pneumonia	*Chlamydia* sp.	N	ND	X	ND
44	Necrotic placentitis	*Stenotrophomonas maltophilia*	N	A	ND	ND
45	Hydrothorax, bacteria in blood vessels of brain and liver	*Escherichia coli*	N	A	ND	ND
46	Necrotic placentitis	*Coxiella burnetii*	X	ND	X	ND
47	Non-specific lesions	*Campylobacter jejuni*	N	C	ND	ND
		*Staphylococcus* sp.	N	A	ND	ND

MZN, Modified Ziehl–Neelsen; IHC, immunohistochemistry; A, Aerobic culture; B, Brucella selective culture; C, microaerophilic culture; F, fungal culture; M, Mycoplasma selective culture; X, positive; N, negative; ND, not done.

Submissions were received from six of nine provinces of South Africa. Farms of origin were mainly in Gauteng province (*n* = 40) followed by North West province (*n* = 30) and Mpumalanga (*n* = 20) (Table 2). *B. abortus* was isolated from bovine submissions from North West, Free State, Mpumalanga and Northern Cape provinces. *C. jejuni* was isolated from ovine and caprine submissions from Gauteng province. *Campylobacter fetus* was isolated from submissions from the Free State and North West provinces. *T. pyogenes* was isolated from bovine cases from Gauteng, North West, Eastern Cape and Free State provinces.

A number of co-infections (*n* = 12) were identified, notably *B. licheniformis* and *Rhizopus* species, *B. abortus* biovar I and *Salmonella* species, *C. abortus* and *Chlamydia pecorum* as well as *Chlamydia* species and Enterobacteriaceae.

Eighteen cases were recorded where there was pathological evidence of an infectious process, but no potential pathogen was isolated. Most cases had placental lesions (*n* = 12), necrotic placentitis (*n* = 6), necrotic placentitis with vasculitis (*n* = 2) or placentitis (*n* = 4).

Additional production-related history (farming system, livestock species, vaccinations, feeding system, herd size, number of abortions) was reported for 50 cases (37%). In 38 of these cases, farming systems were recorded. Farming systems were mostly meat production (*n* = 30) and dairy (*n* = 9). Cattle (*n* = 19) and goats (*n* = 9) were the most common livestock species. In 12 cases, vaccinations were reported. Five of these cases included *Brucella* vaccination. Two cases included *C. abortus* vaccination. Feeding systems were mostly pasture based with supplementary feeding (*n* = 6). One owner reported feeding silage the quality of which was sometimes poor. Nine owners reported the number of abortions and number of pregnant animals or abortion percentage. Abortion percentage ranged from 1% to 75%. Two owners reported abortions over time: nine abortions in five days and four abortions in two days.

Vaccination history was recorded in 12 cases. A wide range of vaccines were used many of which do not have the prevention of abortion as their primary application. Vaccines for the prevention of abortion were *Brucella* S19 and RB51 recorded in five cases, as well as Enzootic abortion vaccine (*C. abortus*) recorded in two cases. Abortions in these seven cases were because of agents other than those vaccinated against indicating that the vaccines were effective or that animals were not exposed to the agents the vaccines are protective against.

## Discussion

In this study, the standard diagnostic approach for abortion investigations in this laboratory, namely impression smears stained by the Gram and modified Ziehl-Neelsen methods, aerobic culture with the addition of *Brucella* culture if requested, was challenged. We investigated the effect of adding anaerobic, *Brucella, Campylobacter, Mycoplasma* and *Leptospira* culture methods and applying the combination of methods to every submission of foetus and placenta samples. The objectives were to determine whether the extended combination of conventional culture methods could increase the recovery of bacterial and fungal causes of abortions. In addition, pathological lesions as well as results of other methods of detection employed by pathologists in case investigations such as PCR and immunohistochemistry (IHC) were recorded.

The number of submissions per year in this study; 83 (2018) and 72 (2019) increased markedly compared to submissions recorded by a retrospective study (2006–2016) by Jonker and Michel (2021) where submissions varied between 11 and 55 per year. The increased number of submissions was probably because of this research project where the conventional bacteriology fees were subsidised. Despite this, submissions were less than the statistically calculated sample size for the study and still biased toward a bacterial or fungal aetiology for the following reasons. Notification of abortion is not compulsory in South Africa. Products of abortion are only submitted for investigation when the field diagnostician and the farmer need a diagnosis, and the farmer can afford the fees. Fees for abortion investigation can be considerable, so cost cutting measures are commonly employed leading to test selection bias. Only samples that are highly suspect for a bacterial or fungal infection will be submitted to a bacteriology laboratory. This approach is contrary to recommendations by Kirkbride (1992) and Borel et al. (2014) that all diagnostic methods available should be employed in every abortion case to ensure the best possible chance of a diagnosis.

Number of submissions decreased as distance increased. Most submissions were received from farms in Gauteng province, which is closest to the Veterinary Faculty at Onderstepoort, followed by neighbouring North West and Mpumalanga provinces. This is probably because of increased costs related to the transport of products of abortion and other veterinary laboratories in closer proximity.

Inclusion of additional methods resulted in two *C. jejuni* isolates that grew only in a microaerophilic atmosphere and would not have been isolated by the previous options for culture. Detection of *Brucella* improved from 7.3% over 10 years (Jonker & Michel 2021) to 7.4% over two years. Anaerobic culture did not yield isolates that could be implicated as causes of abortion. *Leptospira* cultures did not yield isolates. Anaerobes and *Leptospira* are labile and could die before samples reach the laboratory leading to false negative results when culture is attempted. Molecular methods should be investigated as an alternative for the detection of these bacteria as genetic material can still be detected after the death of an organism. Agerholm et al. (2006) reported *F. necrophorum* as the second most common aetiology in their ovine study. In their study, *F. necrophorum* was never cultured but was detected by 16S rRNA gene PCR after bacteria associated with inflammation was noted during the histological examination.

The overall diagnostic rate of 42.2% in this study was higher than the 35.1% found in our previous retrospective study (2006–2016) (Jonker & Michel 2021). Of the cases where a diagnosis was made 82.5% had a bacterial/fungal cause. This was expected because products of abortion are only submitted when a bacterial or fungal infection is suspected. The diagnostic rate for ovine cases (48.0%) was highest followed by bovine cases (32.9%). This phenomenon is probably related to a number of submissions for the different species.

As previously reported, the availability of placenta appeared to positively influence the diagnostic rate. In this study, placentas were received in 37% cases, an improvement over 22.2% of placentas recorded in the retrospective study (Jonker & Michel 2021). This is probably because of continued efforts to emphasise the importance of submission of placenta in reports to clients. However, similar to the retrospective study, no statistically significant association (*p* < 0.05) was found between the overall diagnostic rate and completeness of submission. This is probably because of the small number of cases in these two studies.

The significance of a bacterial isolate is indicated by compatible history, isolation in large numbers in pure or almost pure culture from one or more than one sample, as well as histopathological findings that correlate with the isolate (Anderson et al. 1990; Borel et al. 2014; Kirkbride 1993). Determination of the significance of a fungal isolate is similar to the additional requirement that fungal hyphae must be visualised microscopically in tissues (Borel et al. 2014; Campero et al. 2003). Contamination by bacteria or fungi results in a mixed culture without any dominant isolates (Kirkbride 1986).

*B. abortus* was the most common isolate reported overall, as well as from three provinces: North West, Free State and Mpumalanga. In this study, 7.4% cases were positive for *B. abortus. B. melitensis* was not isolated. Modified Ziehl–Neelsen-stained impression smears were negative in 40.0% of *Brucella* culture-positive cases confirming insufficient sensitivity of MZN-stained smears as reported by Schnydrig et al. (2017). *B. abortus* was also the most common isolate (7.3%) reported previously in South Africa (Jonker & Michel 2021).

In this study, *Campylobacter* species was only isolated in two cases of bovine, one case of ovine and one case of caprine abortion. Only *C. jejuni* could be cultured on CCDA. Although *Campylobacter* species did not appear to play a significant role in abortion the findings clearly highlight the importance of including a nonselective culture medium in *Campylobacter* culture. In contrast with this study, *Campylobacter* species was reported as a significant cause of abortion in countries in the northern hemisphere (the Netherlands and the United Kingdom) as well as Argentina in the southern hemisphere where it was the second most common aetiology in bovine cases (Campero et al. 2003; Carson 2017; Van Engelen et al. 2014). Vaccine can be used to control *C. fetus* subspecies *venerealis* infection. Therefore, it is important to differentiate *Campylobacter* species from one another although phenotypic differentiation of species in this family does present challenges (Van der Graaff-Van Bloois 2014).

*Salmonella* species (5.9%) and *T. pyogenes* (4.4%) were implicated more commonly as agents of abortion compared with the retrospective study by Jonker and Michel (2021). The reason for the increased isolation of *Salmonella* species could be the reduction of test selection bias in this study.

In this study, similar to several other studies, a number of co-infections were recorded, such as *B. licheniformis* and *Rhizopus* species, *B. abortus* biovar I and *Salmonella* species as well as *C. abortus* and *E. coli*. A South African study by Schutte et al. (1976) reported co-infection of *C. burnetii* and *C. psittaci*. Schnydrig et al. (2017) reported 31.2% cases with co-infections. Campero et al. (2003) reported a case of *B. abortus* and *Campylobacter* species co-infection. Agerholm et al. (2006) reported a dual infection of *F. necrophorum* and *L. monocytogenes* in an ovine foetus. The role of bacteria in dual infections needs further investigation as it is not clear whether both agents cause the abortion in such cases.

In 12 cases (8.8%), evidence of an infectious process was seen macro- and/or microscopically, but no agent of abortion was isolated. This is a reduction of cases from 14.6% previously recorded by Jonker and Michel (2021). Application of an extended combination of diagnostic methods to all cases probably contributed to this improvement. More *Brucella* sp. were detected by adding *Brucella* selective culture. *C. jejuni* was detected by microaerophilic culture. *Mycoplasma* species was detected by *Mycoplasma* selective culture. *Salmonella* was detected by aerobic culture in cases where the client only requested *Chlamydia* detection. Increased use of IHC and PCR methods also lead to increased detection of *Chlamydia* sp. and *C. burnetii*.

## Conclusion

- The new combination of conventional culture methods applied to all abortion cases only served to remove test selection bias in the bacteriology laboratory. Nonetheless, the importance of performing an in-depth study on the products of abortion by means of an extensive, combination of conventional culture methods was emphasised by an increase in the number of *B. abortus* and *C. jejuni* isolations. Some of these isolates would have been missed if the culture in a microaerophilic atmosphere and *Brucella* selective culture was not included.
- The combination of methods that yielded the highest number of significant isolates was aerobic, microaerophilic, *Brucella* and fungal culture.

### Recommendations

- Further investigation is recommended to extend the use of such methods to detect and identify difficult to culture agents of abortion to species level.
- *B. abortus* was the most common agent of abortion isolated in this laboratory over a period of two years. Because of the common occurrence of brucellosis, the recommendation is that all laboratories in South Africa employ *Brucella* species detection.
